# The Neurokinin 1 Receptor Antagonist, Ezlopitant, Reduces Appetitive Responding for Sucrose and Ethanol

**DOI:** 10.1371/journal.pone.0012527

**Published:** 2010-09-01

**Authors:** Pia Steensland, Jeffrey A. Simms, Carsten K. Nielsen, Joan Holgate, Jade J. Bito-Onon, Selena E. Bartlett

**Affiliations:** 1 Ernest Gallo Clinic and Research Center, University of California San Francisco, Emeryville, California, United States of America; 2 Department of Clinical Neuroscience, Karolinska Institutet, Stockholm, Sweden; Sapienza University of Rome, Italy

## Abstract

**Background:**

The current obesity epidemic is thought to be partly driven by over-consumption of sugar-sweetened diets and soft drinks. Loss-of-control over eating and addiction to drugs of abuse share overlapping brain mechanisms including changes in motivational drive, such that stimuli that are often no longer ‘liked’ are still intensely ‘wanted’ [7], . The neurokinin 1 (NK1) receptor system has been implicated in both learned appetitive behaviors and addiction to alcohol and opioids; however, its role in natural reward seeking remains unknown.

**Methodology/Principal Findings:**

We sought to determine whether the NK1-receptor system plays a role in the reinforcing properties of sucrose using a novel selective and clinically safe NK1-receptor antagonist, ezlopitant (CJ-11,974), in three animal models of sucrose consumption and seeking. Furthermore, we compared the effect of ezlopitant on ethanol consumption and seeking in rodents. The NK1-receptor antagonist, ezlopitant decreased appetitive responding for sucrose more potently than for ethanol using an operant self-administration protocol without affecting general locomotor activity. To further evaluate the selectivity of the NK1-receptor antagonist in decreasing consumption of sweetened solutions, we compared the effects of ezlopitant on water, saccharin-, and sodium chloride (NaCl) solution consumption. Ezlopitant decreased intake of saccharin but had no effect on water or salty solution consumption.

**Conclusions/Significance:**

The present study indicates that the NK1-receptor may be a part of a common pathway regulating the self-administration, motivational and reinforcing aspects of sweetened solutions, regardless of caloric value, and those of substances of abuse. Additionally, these results indicate that the NK1-receptor system may serve as a therapeutic target for obesity induced by over-consumption of natural reinforcers.

## Introduction

Obesity-related pathology is an alarming public health problem worldwide. Homeostatic control systems precisely regulate body weight and adiposity in a restrictive food environment [1]. However, non-homeostatic factors, such as palatability and motivation, override these systems when a sedentary lifestyle is combined with accessibility of palatable and calorically dense foods or natural reinforcers [1]. In fact, the current obesity epidemic is suggested to be partly driven by over-consumption of natural reinforcers such as sugar [2]–[6].

Uncontrolled over-consumption of natural reinforcers share characteristics with drug addiction. For example, stimuli no longer ‘liked’ are still intensely ‘wanted’ [7], [8]. Additionally, there is overlap between brain regions regulating seeking and self-administration of substances of abuse and those regulating motivational and reinforcing aspects of foraging and intake of natural reinforcers [9]–[15]. For example, natural reinforcers, including sucrose, activate neurons in the ventral tegmental area (VTA). Conversely, VTA lesions selectively reduce sucrose consumption [16]. Furthermore, sucrose consumption increases dopamine release in the nucleus accumbens [17], a brain area exhibiting opiate-like activation following excessive sugar intake [18]. Finally, recent neuroimaging studies have discovered neuroadaptations in obese individuals that mimic those in cocaine addicted individuals [14], [19], [20].

The common molecular substrates underling the motivation to consume natural reinforcers and drugs of abuse are largely unknown. However, the endogenous opioid system is one possible candidate [15]. An interaction between sugar consumption and the opioid system is supported by cross-tolerance [21], [22] and cross-dependence [23]–[25] between sucrose consumption and opiates. In addition, both sugar and ethanol intake is decreased by the opioid antagonist naltrexone [26]–[28].

Recently it has been suggested that the neurokinin 1 (NK1)-receptor system, and its endogenous ligand substance P (SP), interacts with the opioid receptor systems to regulate reward related behaviors. For example, the NK1- and µ-opioid (MOR)-receptors are widely co-expressed in brain regions involved in reward, for example the amygdala, hypothalamus, and nucleus accumbens [29]–[33]. Furthermore, NK1-receptor knock-out mice are insensitive to the rewarding properties of morphine [34], [35]. NK1-receptor knock-out mice fail to develop a preference for morphine, using the conditioned place preference paradigm, and self-administer morphine at lower levels than wild-type controls [35]. Additionally, recent series of preclinical and clinical experiments identified the NK1-receptor as a novel therapeutic target for alcohol use disorders (AUDs) [36]. The preclinical studies showed that NK1-receptor knockout mice decreases voluntary ethanol consumption and increased sensitivity to sedative effects of ethanol [36]. In the clinical setting, the NK1-receptor antagonist LY686017 suppressed alcohol craving and improved overall well-being in recently detoxified AUD patients [36]. The role of NK1-receptor system in AUDs is further supported by a study showing that polymorphisms of the NK1-receptor are significantly associated with the development of AUDs in Caucasian individuals [37].

The involvement of the opioid system in rewarding properties of both sucrose and drugs of abuse, together with the interaction between the opioid and the NK1-receptor system in reward related behaviors led us to the hypothesis that the NK1-receptor system may play a role in appetitive behaviors. To further elucidate the role of the NK1-receptor system in the regulation of consumption of natural reinforcers and ethanol, we evaluated the efficacy of a clinically safe and selective NK1-receptor antagonist, ezlopitant (CJ-11,974) [38], [39] to decrease sucrose and ethanol consumption and seeking. Ezlopitant has previously been investigated in clinical trials as a potential therapy for pain, chemotherapy-induced emesis and irritable bowel syndrome [40], [41]. The present study gives further support to the hypothesis that the NK1-receptor system might be a novel therapeutic target for addictive disorders.

## Results

### The NK1-receptor antagonist ezlopitant decreases both sucrose and ethanol operant-self administration

**Figure 1:**
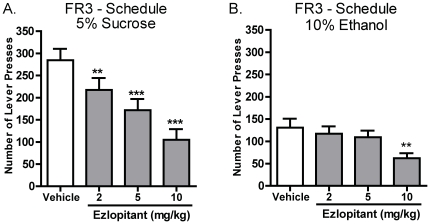
The NK1-receptor antagonist ezlopitant significantly inhibits both sucrose and ethanol operant self-administration in rats. Ezlopitant (2, 5, 10 mg/kg i.p.) dose dependently decreases self-administration of 5% sucrose (**A**). Only the highest dose of ezlopitant (10 mg/kg i.p.) decreases self-administration of 10% ethanol (**B**). All values are expressed as the mean ± SEM number of presses on the active lever. **P<0.01, ***P<0.001 compared to vehicle.

The effect of the NK1-receptor antagonist, ezlopitant on sucrose and ethanol operant self-administration was evaluated in Long-Evans rats that had a stable level of ethanol or sucrose responding on a FR3 schedule. Ezlopitant (2, 5 or 10 mg/kg i.p.) or vehicle was administered 30 minutes before the start of the operant self-administration session. Ezlopitant treatment had an overall main effect on the number of presses on the active lever during operant self-administration of 5% sucrose [F(3,15) = 21.9, P<0.001] and post hoc analysis revealed that all doses of ezlopitant attenuated the number of active lever presses for 5% sucrose (Figure 1A). In addition, ezlopitant treatment had an overall main effect on the number of presses on the active lever during operant self-administration of 10% ethanol [F(3,14) = 5.5, P<0.01]. Post hoc analysis revealed that the highest dose of ezlopitant (10 mg/kg) significantly inhibited operant self-administration of 10% ethanol compared with vehicle (Figure 1B). There was no overall main effect on the number of presses on the inactive lever in the ethanol or the sucrose group [sucrose: F(3,15) = 1.4, non-significant (n.s.); ethanol: F(3,14) = 2.3, n.s., data not shown].

### Ezlopitant inhibits progressive ratio responding for sucrose, but not ethanol

**Figure 2:**
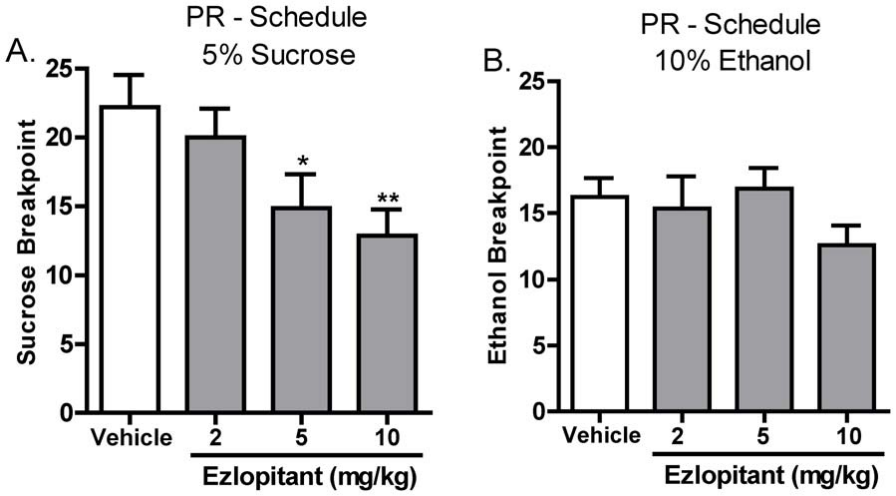
The NK1-receptor antagonist ezlopitant significantly decreases the breakpoint for sucrose but not ethanol operant self-administration in rats. Ezlopitant (5 and 10 mg/kg i.p.) decreases the breakpoint for 5% sucrose using a PR schedule (**A**), but had no effect on the breakpoint for 10% ethanol (**B**). All values are expressed as the mean ± SEM breakpoint. **P<0.01, *P<0.05 compared to vehicle.

The effect of ezlopitant on the incentive motivation to respond for sucrose and ethanol was evaluated in Long-Evans rats that had achieved a stable level of ethanol or sucrose responding on a FR3 schedule. Ezlopitant (2, 5 or 10 mg/kg i.p.) or vehicle was administered 30 minutes before the start of the PR test. Ezlopitant treatment had an overall main effect on the breakpoint during PR tests for the 5% sucrose group [F(3,13) = 5.9, P<0.01] and post hoc analysis revealed that the 5 and 10mg/kg doses of ezlopitant attenuated the breakpoint for 5% sucrose (Figure 2A). In contrast, ezlopitant treatment had no overall main effect the breakpoint during PR tests for the 10% ethanol group [F(4,13) = 1.5, n.s] (Figure 2B). One sucrose animal was excluded from analysis because of low breakpoint responding following vehicle treatment and one ethanol animal was excluded from analysis due to lack of responding during regular FR3 sessions.

### Ezlopitant selectively inhibits voluntary consumption of both sucrose and ethanol

**Figure 3:**
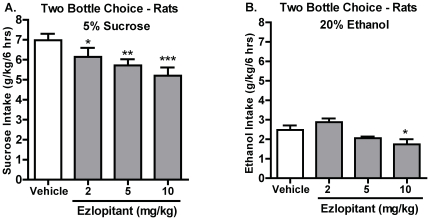
The NK1-receptor antagonist ezlopitant significantly and selectively decreases voluntary intake of both sucrose and ethanol. Ezlopitant decreased consumption of 5% sucrose (**A**), whereas only the highest dose of ezlopitant (10 mg/kg) decreased consumption of 20% ethanol (**B**). All values are expressed as the mean intake (ethanol and sucrose: g/kg/6 hrs) ± SEM. *P<0.05, **P<0.01, ***P<0.001 compared to vehicle.

Ezlopitant treatment had an overall main effect on voluntary consumption of 5% sucrose (g/kg) at both time points [6hrs; F(3,11) = 7.8, P<0.001; 24hrs: F(3,11) = 7.1, P<0.001]. Post hoc analysis showed that all doses of ezlopitant significantly decreased sucrose intake compared to vehicle at the 6 hour time point (Figure 3A). At the 24 hour time point, the two highest doses of ezlopitant significantly decreased sucrose intake compared to vehicle (5 mg/kg: P<0.05 and 10 mg/kg: P<0.001; data not shown). There was an overall main effect on the preference for sucrose over water at the 6 hour, but not the 24 hour time point [6hrs: F(3,11) = 4.0, P<0.05; F(3,11) = 2.1, n.s., data not shown]. Post hoc analysis of the 6 hour preference data revealed a significant decrease following treatment with the highest dose of ezlopitant (10 mg/kg) compared to vehicle (P<0.05, data not shown).

In the high ethanol consumption model (intermittent-access-20%-ethanol), there was an overall main effect on the ethanol consumption (g/kg) 6 and 24 hours after the administration of ezlopitant [6hr: F(3,10) = 8.2, P<0.001; 24hrs: F(3,10) = 5.9, P<0.01). Post hoc analysis revealed that the highest dose of ezlopitant (10 mg/kg) significantly reduced the ethanol consumption compared to vehicle at both time points (6hrs: Figure 3B, 24hrs: data not shown). In addition, there was an overall main effect on the preference for ethanol over water at both time points [6hrs: F(3.10) = 6.9, P<0.01; 24hrs: F(3.10) = 4.2, P<0.05], however, post hoc analysis revealed a significant difference between the highest dose of ezlopitant (10 mg/kg) and vehicle only at the 6 hour time point (P<0.01, data not shown).

The ability of ezlopitant to decrease sucrose and ethanol consumption was specific for each of the solutions as treatment with ezlopitant had no overall main effect on water consumption in either the sucrose or ethanol group at any time point [sucrose group: 6hrs: F(3,10) = 1.4, n.s., Table 1, 24hrs: F(3,10) = 1.8, n.s. data not shown; ethanol group: 6hrs: F(3,11) = 1.3, n.s., Table 1, 24hrs: F(3,11) = 1.0, n.s., data not shown].

**Table 1:** Ezlopitant treatment had no effect on the water intake in rats that were given intermittent-access to 5% sucrose and 20% ethanol, respectively, in a two-bottle choice setting.

Ezlopitant (mg/kg)	Water intake when given access together with:	
	5% Sucrose (n = 12)	20% Ethanol (n = 11)
02510	4.4±0.53.6±0.33.8±0.54.8±0.5	7.1±0.67.3±0.87.3±0.88.5±0.9

The values are expressed as mean water intake (ml/6 hrs) ± SEM.

### The NK1-receptor antagonist ezlopitant decreases voluntary sucrose consumption in C57BL/6 mice

**Figure 4:**
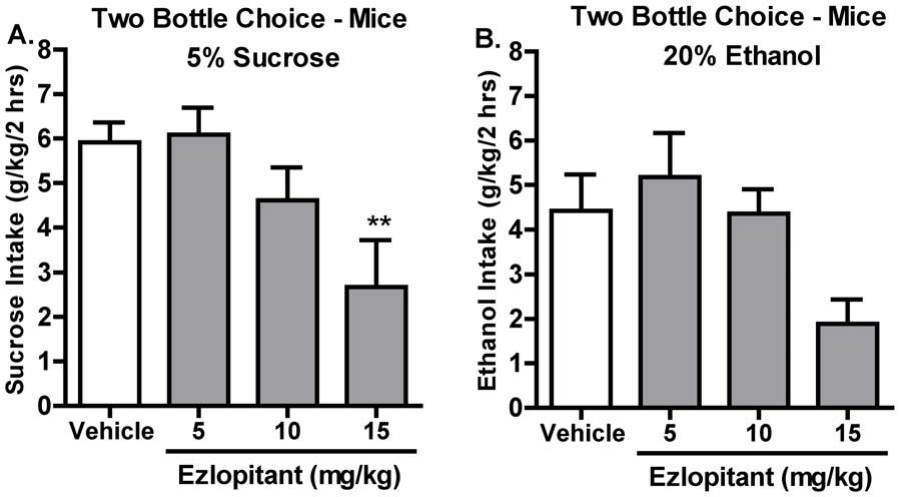
The NK1-receptor antagonist ezlopitant significantly decreases voluntary intake of sucrose using the drinking in the dark model in mice. The highest dose of ezlopitant (15 mg/kg) decreased consumption of 5% sucrose (**A**). However, ezlopitant treatment failed to induce a significant decrease in ethanol consumption compared to vehicle (**B**). All values are expressed as the mean intake (g/kg/2 hrs) ± SEM. **P<0.01, compared to vehicle.

In the DID-model of sucrose consumption, the ezlopitant treatment had an overall main effect on the sucrose intake in C57BL/6 mice [F(3,39) = 4.2, P = 0.01]. Post hoc analysis showed that the highest dose of ezlopitant (15 mg/kg) significantly decreased the voluntary sucrose intake compared to vehicle (Figure 4A). Furthermore, there was an overall main effect on the preference for sucrose over water [F(3,39) = 4.8, P<0.01]. Post hoc analysis revealed that the 15 mg/kg dose decreased the preference for sucrose over water compared to vehicle (P>0.01, data not shown). The decrease in sucrose intake following ezlopitant treatment was specific since there was no overall main effect on the water intake [F(3,39) = 1.1, n.s., data not shown].

In the DID-model of ethanol consumption, there was a trend, but no significant overall main effect on the ethanol consumption (g/kg/2hrs) after ezlopitant administration in C57BL/6 mice [F(3,38) = 2.6, P = 0.06, n.s, Figure 4B]. Subsequently, there was no overall main effect on the preference for ethanol over water following ezlopitant treatment [F(3,38) = 2.2, n.s., data not shown]. Furthermore, there was no overall main effect on the water intake following ezlopitant treatment [F(3,38) = 2.3, n.s., data not shown] in the C57BL/6 mice.

### Ezlopitant does not affect locomotor activity

**Figure 5:**
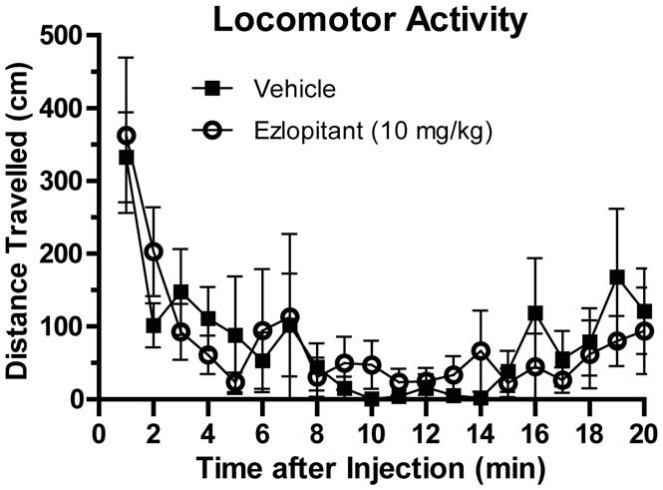
The NK1-receptor antagonist ezlopitant has no significant effect on general locomotor activity in rats. Rats administered ezlopitant (10 mg/kg) did not display differences in ambulatory distances traveled compared to vehicle-treated rats. The values are expressed as the mean (± SEM) distance traveled (cm) per 3 minute-period over the 60 minute test-period.

To examine the possibility that ezlopitant inhibited operant self-administration and consumption of ethanol and sucrose through a general effect on locomotor behavior, we administrated ezlopitant or vehicle to two different groups of Long Evans rats. Following habituation to the locomotor activity boxes, the NK1-receptor antagonist (10 mg/kg) or vehicle was administered and the ambulatory distance traveled was recorded for 60 minutes. The NK1-receptor antagonist treatment induced no significant effect on locomotor activity (ambulatory distance travelled) compared to vehicle (P = 0.92, n.s., Figure 5).

### Ezlopitant selectively inhibits voluntary consumption of the non-caloric sweetener saccharin

**Figure 6:**
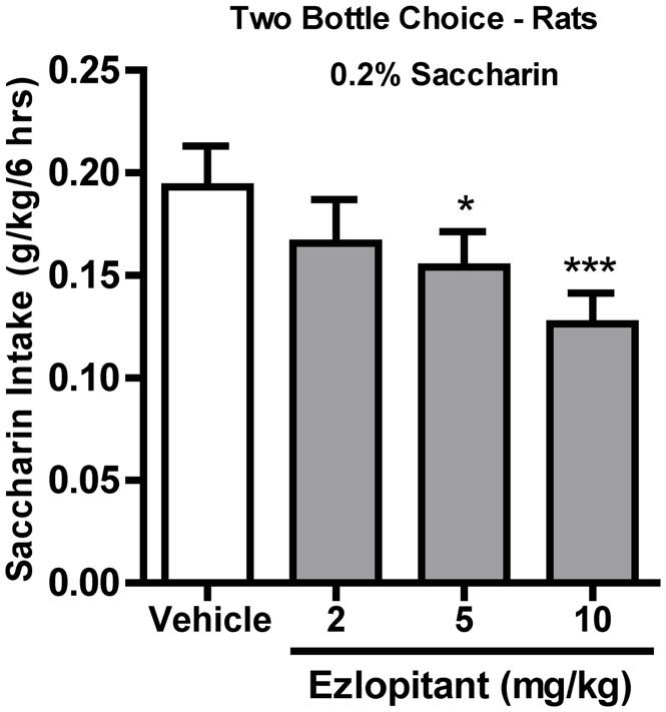
The NK1-receptor antagonist ezlopitant significantly decreases voluntary intake of non-caloric saccharin solution. Ezlopitant (5 and 10 mg/kg) significantly decreased consumption of the non-caloric 0.2% saccharin solution compared to vehicle. All values are expressed as the mean saccharin intake (g/kg/6 hrs) ± SEM. *P<0.05, ***P<0.001 compared to vehicle.

To evaluate if the marked ezlopitant-induced decrease in sucrose compared to ethanol intake was dependent on the high caloric value of the sucrose solution, we tested the effect of the compound on a 0.2% saccharin solution with zero caloric value. Ezlopitant treatment had an overall main effect on voluntary consumption of 0.2% saccharin (g/kg) at both time points [6hrs: F(3,9) = 7.6, P<0.001; 24hrs: F(3,9) = 6.5, P<0.01]. Post hoc analysis showed that the two highest doses of ezlopitant (5 and 10 mg/kg) significantly decreased saccharin intake compared to vehicle at the 6 hour time point (Figure 6). At the 24 hour time point, only the highest ezlopitant dose significantly decreased saccharin intake compared to vehicle (P<0.01; data not shown).

### Ezlopitant does not decrease intake of water or a salty solution

**Figure 7:**
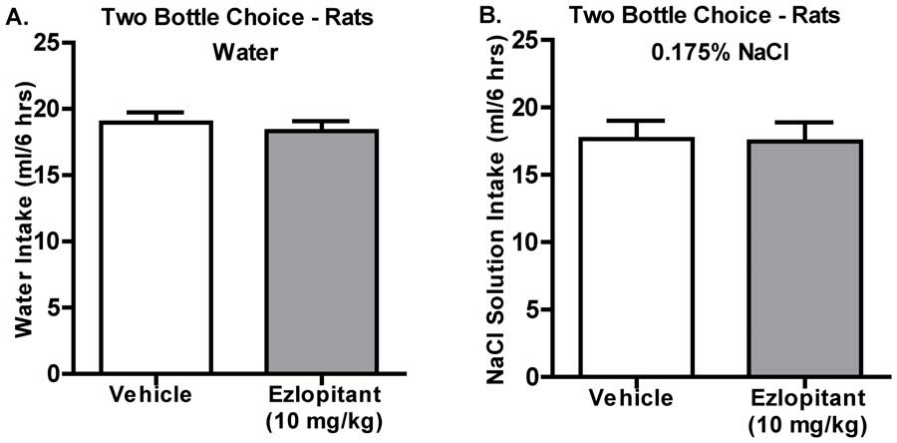
The NK1-receptor antagonist ezlopitant has no significant effect on intake of water or salty solution. Rats administered ezlopitant (10 mg/kg) did not decrease the intake of water (**A**) or a 0.175% NaCl solution (**B**) compared to vehicle. All values are expressed as the mean intake (ml/6 hrs) ± SEM.

To further evaluate ezlopitant's selectivity for sweet solutions, we tested the effect of the NK1-receptor antagonist on water and salty (NaCl) solution intake, respectively. There were no significant difference in water or 0.175% NaCl solution intake at any time point following ezlopitant (10 mg/kg) treatment compared to vehicle (water: 6hrs: P = 0.39, n.s. Figure 7A, 24hrs: P = 0.52, n.s. data not shown; NaCl: 6hrs: P = 0.88, n.s., Figure 7B, 24hrs: P = 0.41, n.s.data not shown).

### The NK1-receptor antagonist ezlopitant inhibits SP-mediated [^35^S]GTPγS-stimulated binding in rat brain membranes

**Figure 8:**
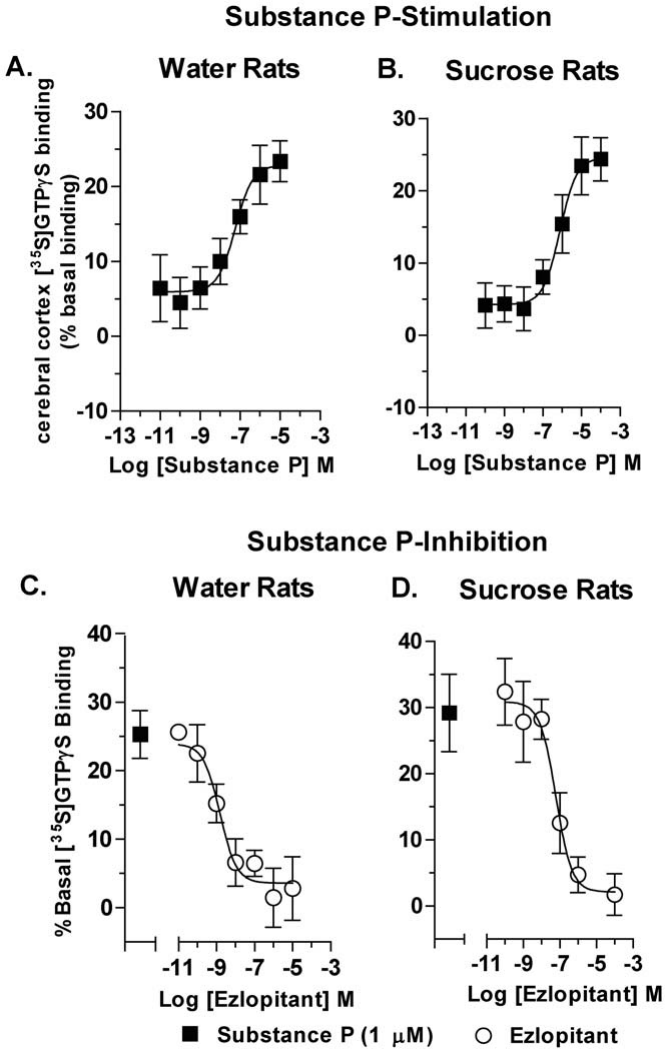
The NK1-receptor antagonist ezlopitant inhibits NK1-receptor-stimulated [^35^S]GTPγS binding in the cerebral cortex of rats. SP produced a dose dependent stimulation of [^35^S]GTPγS-binding in rat membranes from naive (**A**) and sucrose- trained rats (**B**). Ezlopitant potently inhibits SP-stimulated (1 µmol/L) [^35^S]GTPγS NK1-receptor binding in the cerebral cortex of naive rats (**C**) and of rats following long-term sucrose consumption (**D**). The values are expressed as mean ± SEM percentage increase in basal [^35^S]GTPγS binding.

SP produced a dose-dependent stimulation of [^35^S]GTPγS-binding in rat membranes prepared from the cerebral cortex of both water exposed (EC_50_ = 57±3.8 nM, Figure 8A) and sucrose exposed rats (EC_50_ = 750±31 nM, Figure 8B). Furthermore, when the SP-stimulated (1 µmol/L) [^35^S]GTPγS-binding was performed in the presence of ezlopitant (0.1 nmol/L–100 µmol/L) the binding was potently inhibited in both groups (water group, IC_50_ = 1.5±0.5 nM, Figure 8C: sucrose group, IC_50_ = 61±3.1 nM, Figure 8D).

## Discussion

Emerging evidence indicates that the NK1-receptor system is involved in reinforcing mechanisms of drugs of abuse including alcohol. The NK1-receptor has been identified as a possible therapeutic target for AUDs [36] and genetic studies indicate that the NK1-receptor is involved in the etiology of AUDs [37]. The present study gives further support for a role of the NK1-receptor system in appetitive behaviors, as the NK1-receptor antagonist, ezlopitant, inhibits SP binding in rat membranes and decreases both sucrose and ethanol self-administration without decreasing water or salty solution consumption or inhibiting general locomotor activity.

In the present study, the NK1-receptor antagonist ezlopitant is effective in attenuating both sucrose and ethanol intake in rodents. Somewhat surprisingly, ezlopitant more potently inhibited sucrose consumption when compared to ethanol in all drinking models used. While the addictive properties of ethanol are well established (for review see [10], [49]), evidence for food or sugar addiction has been largely based on anecdotal evidence. However, some people claim that they feel compelled to eat sweet foods, similar to how an alcoholic might crave alcohol [9] and it has recently been shown that natural reinforcers stimulate the same neural systems and reward mediating neurotransmitters (including dopamine, acetylcholine and opioids) as ethanol (for review see: [9], [10]). Furthermore, emerging evidence indicate that sucrose is addictive in rodents [9], [18], [23], [50]. Several stages of addiction, for example bingeing [51], withdrawal [9], [23], [52], craving [53]–[55] and cross-sensitization to amphetamine and cocaine [56], [57], can all be induced following intermittent excessive sugar intake. Finally, the reinforcing properties of sugar are supported by a recent study showing that sweetened solutions can surpass cocaine reward, even in drug-sensitized and -addicted rats [58].

Recently, the NK1-receptor antagonist LY686017 was shown to suppress spontaneous alcohol craving in AUD patients [36]. Additionally, the amygdala, which expresses high levels of NK1-receptors, plays a role in the motivational aspects of alcohol drinking behaviors [59]. Thus, LY686017 might attenuate craving by suppression of pathologically elevated activity in the amygdala [36]. Furthermore, MOR and NK1-receptors are co-expressed in amygdala neurons [60] which is an important area for the NK1-receptor's involvement in the motivational aspects of morphine reward [29]. The present results support this hypothesis. In the operant self-administration paradigm, the rats were trained on a fixed ratio (FR3) schedule to obtain an sucrose or ethanol reward and following establishment of stable baseline responding were challenged using a PR schedule. This paradigm is thus used as a measure of the motivation to seek the reward [61]. Ezlopitant was more effective in inhibiting sucrose and ethanol under FR3 operant conditions than in a two-bottle-choice setting where the reward is freely available. Importantly, the decreases in operant behaviors (on both fixed and PR schedules) were more dramatic in the sucrose-trained animals when compared to the ethanol-trained animals. Because, ezlopitant had no effect on general locomotor activity and NK1-receptor antagonists act as antidepressants in rodents [62], the possibility that the ezlopitant-induced decreased activity in the operant self-administration paradigm could be a result of decreased general activity or anhedonia is minimal. Furthermore, ezlopitant significantly attenuated intake of the non-caloric sweetener saccharin, but had no effect on water and salty solution consumption. These results indicate that NK1-receptors play an important role in appetitive responding for sweet solutions, regardless of caloric value, and may regulate the motivational aspects of consumption of ethanol and sweetened solutions.

The present study shows that ezlopitant treatment significantly decreases ethanol consumption in rats, supporting a study showing that NK1-receptor knock-out mice have markedly lower ethanol consumption than wild-type controls [36]. However, in contrast to the rat data, we found that ezlopitant-treatment failed to significantly decrease ethanol consumption compared to vehicle in C57BL/6 mice. One possible explanation is that higher doses of the NK1-receptor antagonist are needed to attenuate ethanol consumption in mice than rats. This is supported by a study indicating that a near-complete inactivation of NK1-receptors is needed to suppress ethanol consumption in mice [36]. Furthermore, in the present study, the lowest dose of ezlopitant (2 mg/kg) significantly decreased sucrose consumption in rats, whereas a markedly higher dose (15 mg/kg) was needed to decrease sucrose intake in mice. These results indicate a species difference in the sensitivity to the behavioral response following NK1-receptor antagonism.

The exact mechanism by which the NK1 receptor antagonist, ezlopitant decreases sucrose and ethanol intake remains unclear. There is strong evidence that both sucrose and ethanol stimulate the brain reward system through endogenous opioids (see for example [10], [11], [13]). The effectiveness of ezlopitant in attenuating sucrose and ethanol consumption indicates there may also be an interaction between NK1-receptors and the endogenous opioid system in reward-related behaviors, but this possibility remains to be determined.

In conclusion, the present study demonstrates that the NK1-receptor antagonist, ezlopitant, decreases sucrose and ethanol consumption and seeking in three different drinking models, giving further support of an involvement of the NK1-receptor system in AUDs and other reward-related behaviors. Additionally, these results indicate that the NK1-receptor system may serve as a therapeutic target for obesity induced by over-consumption of natural reinforcers. A clinical study is possible as ezlopitant is known to be safe in human subjects [40], [41].

## Methods

### Animals and Housing

Male, Long Evans rats (Harlan Indianapolis, IN) and C57BL/6 mice (Charles River Laboratories, Wilmington, MA) were individually housed in ventilated, climate controlled Plexiglas cages. The animals were acclimatized to the individual housing conditions and handling before the start of the experiments. All animals in the two-bottle-choice experiments were maintained on a 12 hour reversed light-dark cycle (lights off at 10 am) and rats in the operant self-administration and locomotor experiments were maintained on a regular 12 hour light-dark cycle (lights on at 7 am). Food and water were available *ad libitum*, except during initial training in the operant self-administration paradigm, as described below.

### Ethical Consideration

The experiments contained herein comply with the current laws of the United States of America. All procedures were pre-approved by the Gallo Center Institutional Animal Care and Use Committee and conducted in accordance with NIH guidelines for the Humane Care and Use of Laboratory Animals.

### Operant Self-Administration Paradigm

#### Fixed Ratio Schedule

One group of Long-Evans rats (233±2 g, n = 16) was trained to self-administer 5% sucrose, and one group of Long-Evans rats (251±4 g, n = 15) was trained to self-administer 10% ethanol using a modified sucrose fading procedure [42] in standard operant conditioning chambers (Coulbourn Instruments, Allentown, PA), as described elsewhere [27]. Both groups were kept on a fixed ratio 3 schedule of reinforcement (FR3; three active lever presses required for 0.1 ml reward, 10% v/v ethanol or 5% sucrose, respectively), daily (Monday through Friday) for 30 minutes for at least five months prior to ezlopitant testing. All rats received all four treatment doses (vehicle, 2, 5 and 10 mg/kg intraperitoneal, i.p.) and each injection was given seven days apart using a Latin square design. Thus, each rat served as its own control.

#### Progressive Ratio Schedule

One group of Long-Evans rats (214±3 g, n = 15) was trained to self-administer 5% sucrose, and one group of Long-Evans rats (238±3 g, n = 15) was trained to self-administer 10% ethanol as described above. Following ∼6 weeks (∼30 sessions) of 30 minute FR3 sessions, both groups of animals were treated with ezlopitant (vehicle, 2, 5 and 10 mg/kg, i.p.) 30 minutes prior to a progressive ratio (PR) test. The PR ratio method is as described by [43]. Briefly, the PR session was initiated by presentation of a compound cue (extension of the levers, illumination of the stimulus light over the active lever, tone sounding, and illumination of a raised dipper cup filled with alcohol or sucrose). After the compound cue, responding proceeded under a PR schedule that was the same for alcohol and sucrose rats. The PR schedule of reinforcement was determined by the equation 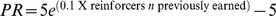. Briefly, after the compound cue, rats could lick the dipper cup, press a lever, or do nothing. If rats licked first, a PR schedule of reinforcement of 1, 1, 2, 2, 3, 4, 5, 6, 7, 9, 10, 12, 13, 15, 17, 20, 22, 25, 28, 32, 36, 40, 45, 50, etc., ensued. If rats pressed first, a PR of 1, 2, 2, 3, 4, 5, 6, 7, 9, 10, 12, 13, 15, 17, 20, 22, 25, 28, 32, 36, 40, 45, 50, etc., ensued. If the rat chose to do nothing, a 20 s timeout period occurred, and the rat was re-cued with the compound cue for up to 20 iterations. For the few rats that required re-cue, one or two re-cues were sufficient to elicit a response. Breakpoint was defined as the number of presses contained in the last, successfully completed ratio in either a 1 hr session or after 15 min of non-responding, whichever came first. Null responses, where an animal completed the required number of lever presses but did not lick to receive the reinforcer, were not counted toward the breakpoint. Ezlopitant-PR tests were performed on Tuesdays and Fridays in a Latin square design with regular FR3 sessions performed the other three days a week. Thus, each rat served as its own control.

### Two-Bottle-Choice Drinking Paradigms

All fluids were presented in 100-ml graduated glass (for rats) or 50-ml plastic cylinders (for mice), with stainless steel drinking spouts inserted through two grommets in front of the cage. Bottles were weighed 6 and 24 hours (for rats) and 2 hours (for mice) after the fluids were presented, and measurements were taken to the nearest 0.1 gram. The weight of each animal was measured daily in order to calculate the gram per kilogram (g/kg) ethanol, sucrose and saccharin intake, respectively. Ethanol and sucrose preference (%) was calculated as the grams of ethanol, or sucrose, consumed divided by the total fluid consumption (grams of ethanol or sucrose+grams of water).

#### Intermittent-Access-20%-Ethanol: High Ethanol Consumption Model

The intermittent-access-20%-ethanol two-bottle-choice drinking paradigm does not require sucrose fading and water is always available *ad libitum* [44], [45]. On the Monday following the end of the housing acclimatization period, 12 Long Evans rats were given access to one bottle of 20% v/v ethanol and one bottle of water. After 24 hours the ethanol bottle was replaced with a second water bottle that was available for the next 24 hours. This pattern was repeated on Wednesdays and Fridays. The rats had unlimited access to water over the weekend after the 24 hour measurement was taken on Saturday morning. After stable baseline drinking levels of 20% ethanol for at least 12 weeks, the rats were administered ezlopitant. All rats received all four treatment doses (vehicle, 2, 5 and 10 mg/kg, i.p.) and each injection was given seven days apart using a Latin square design. Thus, each rat served as its own control.

#### Intermittent-Access-5%-Sucrose

Long-Evans rats (263±5, n = 11) were given intermittent-access to 5% sucrose solution according to the same schedule as the intermittent-access-20%-ethanol model. When rats had maintained stable baseline drinking levels for 12 weeks, administration of ezlopitant began. All rats received all four treatments (vehicle, 2, 5 and 10 mg/kg i.p.) and each injection was given seven days apart using a Latin square design.

#### Intermittent-Access-0.2%-Saccharin

Long-Evans rats (n = 10) were given intermittent-access to 0.2% saccharin solution according to the same schedule as the intermittent-access-20%-ethanol model. When rats had maintained stable baseline drinking levels for ∼5 weeks, administration of ezlopitant began. All rats received all four treatments (vehicle, 2, 5 and 10 mg/kg i.p.) and each injection was given seven days apart using a Latin square design.

#### Continuous-Access to Water

The effect of ezlopitant on consumption of water and salty solution, respectively, was evaluated in the same group Long-Evans rats given intermittent-access to 0.2% saccharin. Following the last ezlopitant-saccharin test occasion, the saccharin was removed and the rats were given continuous-access to two bottles of water. Following a four-day washout period, ezlopitant (10 mg/kg, i.p.) or vehicle was given to the rats to evaluate the effect on water consumption. The treatment was repeated and reversed between animals 48hrs later. Thus, each animal served as its own control.

#### Intermittent-Access-0.175%-Sodium Chloride (NaCl) solution

After the last ezlopitant-water test occasion, the rats were given intermittent-access to one bottle of 0.175% NaCl in water and one bottle of water. Following five days, ezlopitant (10 mg/kg, i.p.) or vehicle was given to the rats to evaluate the effect on salty solution consumption. The treatment was repeated and reversed between animals 48hrs later. Thus, each animal served as its own control.

#### Drinking in the Dark Model

The “drinking in the dark” (DID) model of ethanol consumption (adapted from [46]) induces high levels of ethanol consumption in mice. In brief, male C57BL/6 mice (5–6 weeks of age and 22±0.5g) were given access to one bottle of 20% ethanol and one bottle of water during a two-hour-period (1pm-3pm), Monday to Friday in a reverse light/dark cycle room. Two bottles of water were available at all other times. The weight of each mouse was measured daily to calculate the g/kg ethanol intake. To habituate the mice to injections, one saline injection (10 ml/kg body weight) was given on two consecutive days, a week before the start of the treatment. Following stable baseline consumption during six-seven weeks (30–38 drinking sessions, 5.0±0.2 g/kg/2 hrs), the mice were randomly assigned to four different doses of ezlopitant (vehicle (n = 12), 5 (n = 8), 10 (n = 15) or 15 (n = 7) mg/kg i.p).

The DID method was also used as a model of sucrose consumption. Male C57BL/6 mice (5–6 weeks of age and 19±0.5g) were given access to 5% sucrose instead of ethanol and subjected to ezlopitant treatment and the same habituation schedule as described above (vehicle (n = 15), 5 (n = 10), 10 (n = 12) or 15 (n = 6) mg/kg i.p).

### General Locomotor Activity

Locomotor studies were run in activity-monitoring chambers (40×40 cm) with horizontal photo beams (Med Associates, St Albans, VT). Horizontal locomotor activity was monitored at 100 ms throughout the sessions. The study was run in 4 daily 2-hour-sessions as described previously [47]. In brief, after habituation of boxes (Day 1) and injections (Day 2 and 3) testing was conducted on Day 4. Data from Day 3 was used to assign animals to one of two treatment groups (vehicle, or ezlopitant (10 mg/kg, i.p.), n = 6 per group). After 60 minutes, a single injection of the assigned treatment was given, subsequently, the session continued for an additional 60 minutes. Data was collected across the entire 2-hour-session and recorded as distance traveled in cm.

### [^35^S]GTPγS Binding in Rat Membranes

Single drug dose-response curve of Substance P (SP)-stimulated (0.1 nmol/L–100 µmol/L) [^35^S]GTPγS-binding and SP-(1 µmol/L)-stimulated [^35^S]GTPγS-binding in the presence of ezlopitant (0.1 nmol/L–100 µmol/L) were performed in triplicate in membranes prepared from rat cerebral cortex as described previously [48]. The brain tissue was collected from water rats (n = 3) as well as from rats that had consumed 5% sucrose (n = 3) according to the intermittent-access schedule described above. [^35^S]GTPγS-stimulated binding was assessed with NXT TOPCOUNTER and expressed as a percentage increase in basal [^35^S]GTPγS-binding.

### Drugs and Chemicals

[^35^S]-guanosine 5′-(γ-thio)triphosphate ([^35^S]-GTPγS) (250 µCi; 9.25 MBq) (Perkin-Elmer, Boston, Massachusetts) and SP (Sigma Aldrich, St. Louis, MO, USA), other chemicals used in binding assays supplied as described previously [48]. All solutions were prepared in filtered water from 95% (v/v) ethanol (Gold Shield Chemical Ac., Hayward, CA, USA), (w/v) sucrose (Fisher Scientific, NJ, USA), saccharin or NaCl (Sigma Aldrich, St. Louis, MO, USA), respectively. The NK1-receptor antagonist, ezlopitant (CJ-11,974) [(2S,3S-cis)-2-diphenylmethyl)-N-[(2-methoxy,5-isopropylphenyl)methyl]-1-azabicyclo-[2.2.2]octan-3-amine)], was generously provided by Pfizer Global Research and Development, Groton, CT, USA. The compound was prepared in saline immediately before each injection. All injections were given as an acute i.p. injection (1 ml/kg for rats and 10 ml/kg for mice), 30 minutes before bottles were presented or before the start of the operant self-administration session.

### Statistics

Statistical analysis was performed using GraphPadPrism software. Data were analyzed by one way ANOVA (mouse data) or repeated measures ANOVA (rat data) followed by Newman-Keuls Post hoc analysis when a significant overall main effect was found (*P*<0.05). The locomotor, water- and NaCl-2-bottle-choice data were analyzed with Student's t-test. Data from *in vitr*o functional binding assays were analyzed by non-linear regression with a sigmoidal curve with variable slope to determine EC_50_ and IC_50_ values.
